# An interview with Kotaro Tanimoto

**DOI:** 10.1590/2177-6709.21.4.024-033.int

**Published:** 2016

**Authors:** Emanuel Braga, Fernando Habib, Márcio Lisboa

**Affiliations:** 1 » Professor of Orthodontics and Pediatric Dentistry, Universidade Federal da Bahia (UFBA), Salvador, Bahia, Brazil. » PhD in Orthodontics, Hiroshima University, Japan. » Vice-president, Association of Former Research Fellows Brazil/Japan (ABRAEX), Brasília, Distrito Federal, Brazil.; 2 » Professor of Orthodontics, Universidade Federal da Bahia (UFBA), Salvador, Bahia, Brazil. » PhD in Dentistry (Laser Therapy), Universidade Federal da Bahia (UFBA) and Universidade Federal da Paraíba (UFPB). » Specialist in Orthodontics, Universidade Federal do Rio de Janeiro (UFRJ), Rio de Janeiro, Rio de Janeiro, Brazil. » Coordinator, Specialization course in Orthodontics, Universidade Federal da Bahia (UFBA), Salvador, Bahia, Brazil.; 3 » Professor of Prosthesis and Occlusion, Universidade Federal da Bahia (UFBA), Salvador, Bahia, Brazil. » PhD in Dentistry (Laser Therapy), Universidade Federal da Bahia (UFBA) and Universidade Federal da Paraíba (UFPB). » Coordinator of Núcleo Innovare de Educação.

**Figure:**
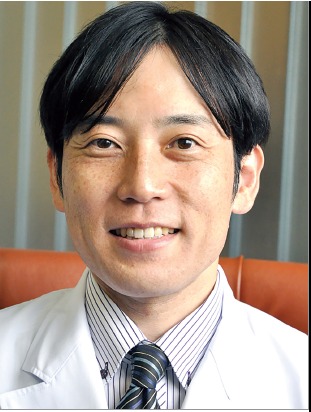

If I had to describe Tanimoto Sensei using a single word I would say kindness. When I was attending the PhD course in Hiroshima University, I frequently looked at him and thought: "If I have the chance to be an educator, I would like to be like that man." The generosity, respect and friendship he employed to treat the students is fascinating. By that time, Professor Tanimoto had just come back from his post-doc in the USA full of ideas and dreams. Since that, many of the ideas had become projects and many of the projects had become a real benefit for the patients. Tanimoto Sensei has an extensive and relevant scientific production and some of them are described in the next pages. I believe this interview is especially interesting because it is enriching to know how professors from the other side of the globe conduct orthodontic education and science. Finally, I would like to express my sincere gratitude to the other professors that assisted in this article and to Dental Press Journal of Orthodontics for the opportunity to conduct the present interview with Professor Tanimoto, a great friend of mine. 

Emanuel Braga - interview coordinator 

Se eu tivesse que descrever Tanimoto Sensei em apenas uma palavra, eu diria "bondade". Na época em que eu estava cursando o doutorado na *Hiroshima University*, com frequência eu olhava para ele e pensava: "*Se eu tiver a oportunidade de me tornar um educador, eu gostaria de ser como aquele homem*". A generosidade, o respeito e a amizade com os quais ele tratava seus alunos eram fascinantes. Naquela época, o professor Tanimoto tinha acabado de voltar de seu pós-doutorado nos EUA, cheio de ideias e sonhos. Desde então, muitas de suas ideias se transformaram em projetos e muitos de seus projetos se tornaram benefícios para os pacientes. Tanimoto Sensei tem uma produção científica extensa e relevante, e alguns de seus trabalhos estão descritos nas próximas páginas. Acredito que esta entrevista seja muito interessante, pois é enriquecedor saber como professores do outro lado do mundo conduzem a formação e ciência ortodônticas. Por fim, eu gostaria de expressar a minha sincera gratidão aos outros professores que contribuíram para este texto e ao *Dental Press Journal of Orthodontics*, pela oportunidade de coordenar a presente entrevista com o professor Tanimoto, um grande amigo meu. 

Emanuel Braga - *coordenador da entrevista*

## Taking Brazil location as a reference, Japan is placed exactly on the other side of the globe. I usually joke that if you are flying from Brazil to Japan and you miss the landing place, you are automatically getting closer to Brazil again. Japan and Brazil are separated by a half planet and many differences are undoubtedly expected. Regarding education, Japan has a brilliant experience to share and I think many Brazilians are interested in knowing how dental education is performed there. Dear Tanimoto Sensei, could you please make a brief description about how orthodontic undergraduate education works in Hiroshima University? Emanuel Braga

In Japan, undergraduate dental education is usually designed as a 6-year program. The program in Hiroshima University School of Dentistry is characterized by several unique systems, such as the Two-Course System, BioDental Education, and Dual-Linguistic Education. Currently our school has a 2-semester system, and the Orthodontics lecture is held for one year from the second semester of the 3^rd^ grade to the first semester of the 4^th^ grade (22 times total). In addition, Stomatognathic Function and Periodontology regarding Orthodontics are also lectured. Thereafter basic practice of Orthodontics is held in the second semester of 4^th^ grade (15 times total). 

In Hiroshima University School of Dentistry, the Two-Course system, a quite unique system in Japanese dental schools, is given from the second semester in the 3^rd^ grade to the first semester in the 5^th^ grade. Undergraduate students in the 3^rd^ grade can select "Course for Frontier Science" or "Course for Clinical Dental Science." In the "Course for Frontier Science," students learn basic science through investigation according to a topic given to each student. On the other hand, the "Course for Clinical Dental Science" has advanced clinical lectures and practices, such as Typodont practice in Orthodontics. Finally, 1-year clinical practice starts from the second semester of the 5^th^ grade.

The undergraduate dental program in Hiroshima University has been continuously reorganized under the concept of BioDental Education and Research since 2010. BioDental Education is designed as a novel dental curriculum to train professionals with scientific background capable of innovating the next generation dental medicine through specific educational programs. The programs are accomplished according to attainment target type education, problem-based learning and research methodology. Scientific knowledge is quite important to every dentist, and training of dental educators with high research background has been an urgent issue in many countries. To date, the nurturing of dental research has been performed mainly in graduate programs in Japan; however, the number of dental students willing to attend graduate courses is not high enough. In addition, few graduates continue research after completion of the graduate course. Due to this situation, we consider that the upbringing of scientific motivation needs to be integrated with undergraduate dental education.

Since another aspect of BioDental Education is to correspond to globalization, Hiroshima University School of Dentistry established the International Dental Course (IDC) in 2011. IDC accepts undergraduate dental students from partner universities (Airlangga University in Indonesia, University of Medicine and Pharmacy in Ho Chi Minh City in Vietnam, and University of Health Sciences Cambodia in Cambodia in 2016). IDC students join our undergraduate programs from the 2^nd^ grade to the first semester of 5^th^ grade after completion of their first year education in their home countries. All lectures and practices during this period are given in both English and Japanese (Dual-Linguistic Education). The purpose of IDC is not only to provide English education, but also stimulate learning motivation and speed up future communication among various countries.

## During my PhD in Hiroshima University, I was amazed by how well balanced clinical training education and research work in the graduate program were. In my opinion, all students had the chance to become well-trained orthodontists and brilliant scientists. Could you please talk about the orthodontic PhD course in Hiroshima University? Emanuel Braga

After graduating from dental school, most of Japanese dental students receive clinical training as residents in a specified dental hospital; thereafter some of them attend the graduate course. Hiroshima University graduate course for PhD in the Department of Orthodontics consists of several special orthodontic lectures/practices and research practice. Graduate students have to complete all of them. The graduate orthodontic education is designed to complete the attainment targets for orthodontic specialist certification managed by the Japanese Orthodontic Society (JOS). The JOS guidelines for orthodontic specialist certification require at least a 5-year orthodontic training, consisting of a 2-year or 3-year basic training in a certified training facility. At present, in Japan, there are 31 training facilities in 29 universities including our department. The orthodontic training program in each facility follows the JOS guidelines, and Biological Science is included in the attainment targets. Therefore, the graduate course in our department provides both orthodontic clinical and research training programs. The orthodontic clinical training consists of Typodont practice, cephalometric analysis, diagnosis of temporomandibular disorders (TMD) by means of MRI, CT-based tridimensional analysis, caries risk examination, periodontal care, stomatognathic examination, electromyogram examination, diagnosis of respiratory and speech function, an so on. Applicants are required to contribute with more than 150 cases and finish at least 30 of them. 

Furthermore, PhD students receive research training, such as research ethics, research planning, experiment techniques, grant application, management of research equipment and material, presentation, and writing. An individual research theme is given to each PhD student, and they have to complete their research to obtain a doctor's degree. Current main research projects in our department are (1) elucidation of pathogenic mechanism in malocclusion and management of oral maxillofacial skeletal growth; (2) optimization of tooth movement by orthodontic forces; (3) establishment of a new orthodontic treatment based on the image and biochemical diagnosis of temporomandibular disorders (TMD); (4) bone regeneration by use of mesenchymal stem cells and the development of regeneration medicine for closure of jaw cleft; (5) elucidation of pathogenesis of amelogenesis imperfecta and the restoration of tooth enamel by application of biomineralization processes; (6) evaluation of the contribution of various oral functions to general fitness; and (7) development of new instruments and material for orthodontic treatment.

Tight collaboration with other departments, such as Oral Pathology, Oral Biochemistry, Oral Anatomy, Biomaterial Technology, and Bacteriology, enables us to correspond to various research fields from basic to clinical research.

## In Hiroshima University, first-year students have to come one hour earlier in order to clean and prepare the rooms for the other members of the Department. In the beginning, I found this task strange and exhausting, especially because PhD students in Brazil usually do not do that. When I became a second-year student, I realized that I was completely unable to throw any piece of garbage in the wrong place. I will never forget this experience. What do you have to say for societies which do not have this sense of community as strong as in Japan? Emanuel Braga

Since graduate students are qualified professional dentists as well as students, they need to have common sense and manners as members of society. There is a Japanese proverb that says: "Start with a bow and end with a bow." Although there are professional sweepers in our university, new members of the department have to clean the department rooms by themselves. As you have mentioned, it may be very difficult to understand sweepers' work and have sincere gratitude for them unless we experience the exact same work. In Japan, having common sense and manners as a member of society are generally reinforced in companies after employment; whereas there is no such opportunity for most dentists, especially for graduate students. Therefore, we intentionally give them such duties just for one year. This way may be the Japanese traditional style, but although there are various senses of values in our society, they are not understood everywhere in our country. Since there are various social systems with different manners and rules in different countries, I think there can be many ways of education.

## Dear Professor Tanimoto, I am very interested in your studies about bone regeneration in cleft sites by means of mesenchymal stem cells and carbonated hydroxyapatite particles. What is the current status of this research and what is the protocol for bone grafting in cleft patients in Hiroshima University? Fernando Habib

I really appreciate your interest in our research regarding bone regeneration for the treatment of cleft palate. Secondary bone graft for patients with cleft palate is usually performed around 8 to 10 years of age, just before canine eruption. Currently, nearly all patients with cleft palate receive iliac bone graft. However, iliac bone graft, especially its classic large-incision, open techniques for bone graft harvesting from anterior and posterior parts of the iliac crest, is surgically invasive, leading to a large encumbrance for patients. Common donor site complications for the anterior ilium are pain, sensory loss, gait disturbance, infection, and hematoma formation. In addition, it is occasionally difficult to obtain enough volume for transplantation in young patients. 

Tissue engineering with transplantation of mesenchymal stem cells (MSCs) derived from bone marrow is a possible candidate to achieve osteogenesis in bone defects and reduce the surgical invasiveness incident associated with the extraction of transplant. The bone marrow can be obtained by aspiration using a bone marrow puncture needle with minimum surgical invasion compared with the separation of iliac bone. Although MSCs constitute only 0.001-0.01% of the subcellular components in bone marrow, cultured bone marrow-derived MSCs can proliferate without losing the potential to differentiate into multiple mesenchyme lineages, such as chondrocytes, adipocytes, and osteoblasts, with corresponding biological stimuli. Therefore, we started the study for regeneration medicine using bone marrow-derived MSCs for treatment of cleft palate.

**Figure 1:**
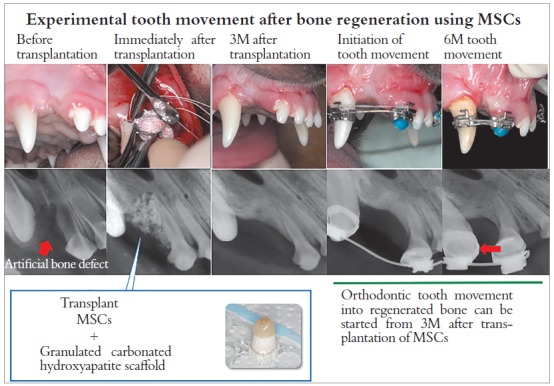
Experimental tooth movement after bone regeneration using MSCs (Source: Tanimoto et al,^1^ 2015).

Among many factors believed to be crucial for tissue regeneration, the scaffold for alveolar bone regeneration in cleft palate patients must possess unique properties. Under the general environment in the oral cavity, various mechanical forces, such as tongue pressure and biting force, are always loaded onto the transplanted area, leading to deformation or leakage of transplants. Therefore, it is necessary for the scaffold to have sufficient strength against external forces without any coverage with hard material over the soft scaffolds, such as collagen gel. For the treatment of bone defect of cleft palate, it is essential to guide or move tooth into the regenerated bone area. It is thus required that the alveolar cleft area after regeneration is filled with new bone with normal metabolism crucial for tooth movement and subsequent maintenance of tooth. Based on these considerations, the scaffolds for bone regeneration in cleft palate patients should be digested quickly. Carbonated-hydroxyapatite (CAP) is originally a major mineral constituent of bone and teeth. It is highly substituted with 3-5% carbonate ions instead of phosphate ions compared with the lattice of hydroxyapatite, leading to an unstable crystal structure with high solubility compared with that of hydroxyapatite. We examined bone regeneration with bone marrow-derived MSCs and CAP scaffold for artificially created alveolar cleft in beagle dogs (Fig 1). The transplantation of MSCs with granulated CAP scaffold induced bone regeneration in artificially created alveolar cleft and was followed by tooth movement to the regenerated bone with a consistent rate of tooth movement. 

So far, the above results were obtained using an animal model. Currently, we are going to start clinical trials as the next step. The Translational Research Medical Center was established in the newly built Hiroshima University Hospital outpatient clinic in 2013. The center has cell processing rooms and equipment for regeneration medicine. Continuous basic and clinical research is still indispensable to carry out bone regeneration medicine for cleft palate in the future.

## Professor, Hiroshima University has played a relevant role regarding basic research for regeneration medicine over the years. In this context, I would like to highlight your research using amelogenin, an enamel protein, which has proved effective for bone regeneration in several circumstances. How is this topic evolved? Is there any clinical use already established? Fernando Habib

Amelogenins are enamel matrix proteins that mainly contribute to tooth enamel formation. Such hard tissue formation by specific proteins in organisms is generally called biomineralization. I conducted a study on biomineralization for enamel formation when I studied abroad at UCSF.

However, enamel matrix proteins have been recently suggested to exert various biological activities. Thus, we focused on biological effects of amelogenins on bone marrow-derived MSCs. MSCs have the ability to differentiate into several types of cells, such as osteoblasts, and we found that full-length amelogenin enhances osteogenic differentiation of MSCs. Accordingly, we considered that full-length amelogenin might be useful for regeneration medicine, especially bone regeneration. However, full-length amelogenin is a highly hydrophobic protein with high molecular weight. Consequently, it easily precipitates in solutions with neutral pH. That means that full-length amelogenin might not always exert intended biological activities in a vital body.

For this reason, we started to research critical amino acid sequences with physiological activity of full-length amelogenin. Since amelogenin is cleaved into small fragments by specific proteases after secretion from ameloblasts into enamel matrix, we speculated that some of these fragments with active domain remain with biological functions. We examined the physiological activity of synthesized potential amelogenin fragments and specified a critical amino acid sequence. The peptide consists of 11 amino acids derived from full-length amelogenin (AMG-peptide) which exerts an activity almost equal to osteogenic differentiation of MSCs, as compared to full-length amelogenin. AMG-peptide can be industrially synthesized in bulk and easily dissolved in water due to high hydrophilic properties. Meanwhile, we found a problem: AMG-peptide is quite easy to diffuse in vital tissues after administration, which suggests that it is difficult to exert its physiological activity sustainably. To solve this problem, we fixed AMG-peptide onto a solid surface to prevent diffusion. We examined whether or not AMG-peptide activity still remains even after fixation to a solid surface. AMG-peptide stably kept its activity. 

For clinical application, AMG-peptide can be fixed onto a scaffold surface to enhance bone formation, or support guided bone regeneration using an AMG-peptide-fixed barrier membrane. Although clinical application is currently not attained, AMG-peptide is expected as one of the potential candidates for safe and effective bone regeneration.

## An interesting topic investigated at Hiroshima University is cryopreservation. Professor, could you talk about cryopreservation and Orthodontics, especially about the benefits of cryopreservation in surgical cases? Fernando Habib

We have studied about long-term cryopreservation of teeth. This has already been made practical, and the teeth cryopreservation service has been provided to patients. During the freezing process of vital tissue, ice crystals generated in the cell body fatally damage cells. Therefore, it is quite important to suppress ice crystal formation and its growth, so as to minimize cell damage as much as possible. For this purpose, both temperature management to quickly pass the freezing point of water during the freezing process and the component of stock solution are critical. Right now the optimal condition for tooth cryopreservation is controlled by means of a specially programmed freezer and stock solution. Cryopreserved teeth can be used for tooth transplantation in case the patient accidentally loses their teeth. 

 In addition, cryopreserved stem cells can be used for tissue regeneration. Currently, we are investigating bone regeneration using dental pulp tissue-derived stem cells (DPSC) from permanent or primary teeth. DPSC can be easily obtained from extracted teeth for orthodontic treatment or by shedding primary teeth, and cryopreserved DPSC can be used for various purposes in the future. DPSC cryopreservation is expected to become a new medical service by consociation with dental clinic.

## Previous studies with the finite element method indicate that disc displacement could affect stress distribution on the condylar articular surface during prolonged clenching. In adult patients with clenching and disc displacement, can occlusal plane splints be helpful? Is there any advantage to use the splint at night only or all day long? Márcio Lisboa

I greatly appreciate you for your question regarding our research and clinical procedure for temporomandibular disorders (TMD). At our department, we occasionally use the stabilization-type splint for TMD patients with unstable occlusion due to malocclusion, so as to diagnose the presence of bruxism and its frequency and time lapse.

For manufacture and application of any type of occlusal splint, we follow the guidelines established by the Japanese Society for the Temporomandibular Joint. The stabilization-type splint is classified in upper dentition type and lower dentition type, and we usually use the former. The stabilization-type splint can be used for equation of mechanical stress applied to the temporomandibular joint (TMJ) between the right and left side. In such cases, it is important to use the splint at the time when patients are clenching their teeth. If timing is unclear, patients are encouraged to start splint therapy at night. The dentist in charge carefully checks for changes in clinical symptoms and abrasion of splint surface; if no change can be observed, patients start using the splint during the day. Either way we prohibit patients to use the splint continuously for more than 15 hours, so as to prevent irreversible occlusal changes due to mandibular shift and/or tooth movement.

 Recently, it has been suggested that tooth contact habits on daytime may cause TMD even if each contact force is relatively light, and the patients are unaware of their own habits in most cases. The effects of the stabilization-type splint on the human body differ between limited short-term application for several weeks at night and continuous long-term application for several months on both daytime and night. In our clinic, short-term splint therapy is selected for most patients. The purposes of the short-term splint therapy are to relief excessive mechanical stress on TMJ and masticatory muscles and to suppress stomatognathic parafunction. Furthermore, reduction of traumatic pain due to excessive occlusal force and placebo effect are also expected. The stabilization-type splint is mostly used for TMD diagnosis and for relief of initial TMD symptoms before orthodontic treatment, and the splint therapy is usually completed when TMJ symptoms are ameliorated. In case of prolonged clenching or if bruxism remains at night, splint therapy is extended as necessary. On the other hand, if any treatment effect can be observed even after splint therapy for several weeks, treatment is immediately finished.

## The articular disc appears to be crucial in the regeneration of a damaged condyle, suggesting that defects or damage to the articular disc may influence mandibular growth and regeneration or repair of the condyle. In this context, teenagers with disc displacement with and without reduction may have problems with the TMJ and occlusion in adulthood? Márcio Lisboa

**Figure 2:**
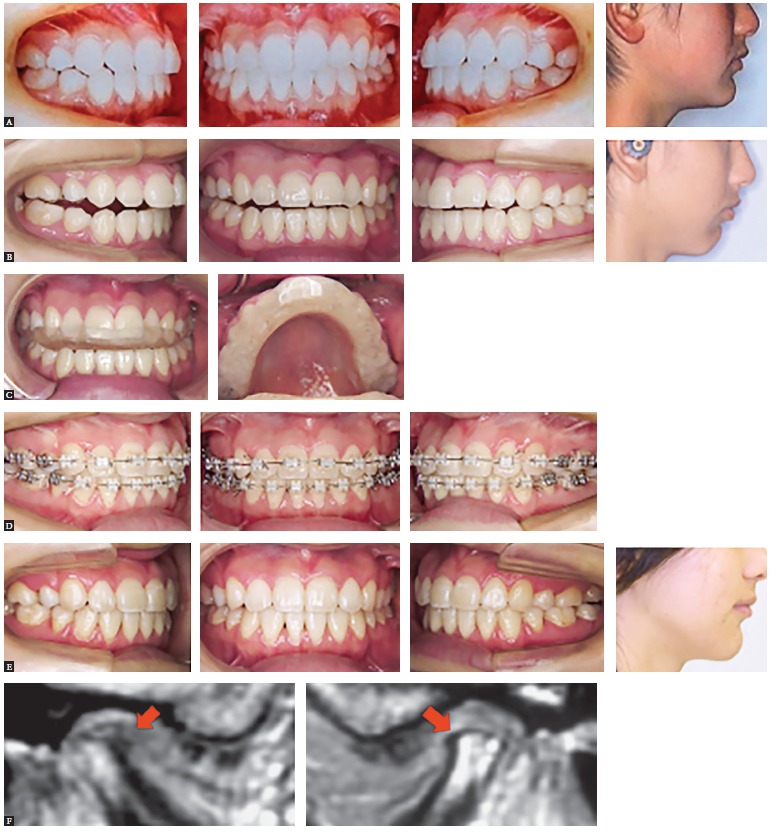
A treatment case with open bite accompanied by osteoarthritis of the temporomandibular joint. A) Initiation of retention at a previous hospital. B) Initial arrival at our hospital. C) Initiation of splint therapy. D) Orthodontic treatment with multi-bracket appliances. E) Removal of all appliances. F) MRI indicated TMJ-osteoarthritis on both sides at initial arrival (Source: Tanimoto^2^, 2011).

Disc displacement does not necessarily cause serious adverse effects on mandibular condylar growth. However, TMJ osteoarthritis (TMJ -OA) in young patients occasionally causes severe changes in occlusion and facial configuration, such as skeletal open bite, mandibular shift, and mandibular retrusion. Figure 2 shows a treatment case of open bite caused by TMJ-OA. Surgical orthodontic treatment is also frequently selected to treat these severe malocclusions. A sufficient observation period is indispensable before initiation of orthodontic treatment, and close attention must be paid to pathologic status of TMD during treatment. Furthermore, a long-term retention period is desirable after orthodontic treatment. In our clinic, all orthodontic patients with TMD undergo TMJ-MRI examination. Since nearly all patients with mandibular condylar resorption are accompanied by disc displacement, these findings seem to have some relation. Disc displacement might be no more than a symptom of degenerative TMJ disease, such as OA.

## The Gnathology Society showed the role of occlusion in Dentistry. Nowadays, what is the importance of occlusion to TMD? Márcio Lisboa

Occlusion is no more than one factor among various causes of TMD, and its contribution to TMD is not so strong as previously believed. There are many severe malocclusion patients without TMD. So far, the prevalence of TMD in orthodontic patients, at least in our clinic, is not very different from that of the general population. Major causes of OA and internal derangement are believed to be excessive forces applied to TMJ, whereas internal causes, such as changes in the property of synovial fluid, are also suggested. 

Our previous study indicated that intra-articular inflammation induces degradation and decreased synthesis of lubricants in joint fluid, such as hyaluronan (HA) and superficial zone protein (SZP). Due to this process, homeostasis of synovial fluid components cannot be maintained, thereby resulting in deterioration of lubricating and buffering functions in the joint. Disc displacement can be induced under these pathological changes of intra-articular environment. If this condition is kept in the long term, severe degeneration or resorption of articular cartilage and subchondral bone are likely to be caused.

**Figure 3:**
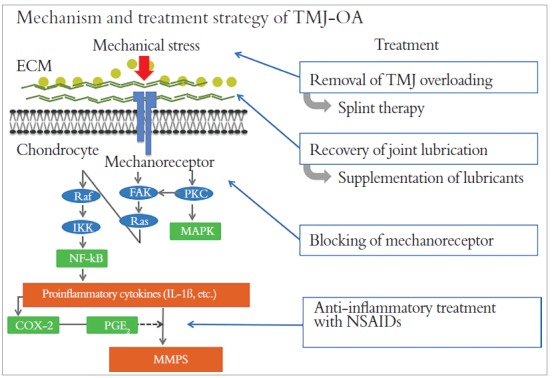
Mechanism and treatment strategy of TMJ-OA.

As of this time, there is no way to completely control the pathological status of OA; therefore, we attempt to establish a phased strategy for OA treatment based on basic research results (Fig 3). In most cases, initial treatment consists of removing excessive mechanical stress from the TMJ, for instance, elimination of oral habits and splint therapy. Second, the recovery of lubricating and buffering function of the synovial fluid by maintaining the intra-articular environment is considered, for instance, by the use of HA supplements or other lubricating molecules by intra-articular injection. A previous research of ours revealed the effectiveness of SZP administration, so as to recover joint function, especially the boundary lubrication ability. In addition, the blocking of mechanoreceptors and administration of NSAIDs are investigated in parallel as treatment protocols for severe conditions.

## It is known that good orthodontic treatment outcomes lead to TMJ health. In your opinion, is it appropriate to treat orthodontically patients with TMD muscle or articular symptomatology? Márcio Lisboa

The important thing for establishing good TMD treatment goals is not occlusion itself, but to manage present disharmony of masticatory muscles and overload onto the TMJ. Outer forces enough to have a bad influence over TMJ hard-tissue remodeling as well as internal causes would become a trigger of pathogenesis. Therefore, we attempt to carefully examine TMJ pathology and dysfunction before starting orthodontic treatment, but never plan it for TMD treatment as a main purpose. If any symptom of TMD is observed in orthodontic patients, differential diagnosis for TMD is performed after careful examination. According to the Japanese Society for the Temporomandibular Joint, TMD is roughly classified into four types: disorders by masticatory muscle pain, disorders by TMJ pain, disorders by disc displacement, and OA. Management of TMD is determined according to each classification. Orthodontic treatment is never initiated if patients have acute symptoms or progressive pathology of TMJ. Adequate initial TMD treatment followed by a sufficient observation period is usually considered in advance. Particularly, if TMD patients have oral habits, such as bruxism, it would also affect the stability of occlusion after orthodontic treatment. 
